# Use of mouse primary epidermal organoids for USA300 infection modeling and drug screening

**DOI:** 10.1038/s41419-022-05525-x

**Published:** 2023-01-11

**Authors:** Xiaorui Xie, Xuebo Tong, Zhihong Li, Quan Cheng, Xiaowei Wang, Yin Long, Fangbo Liu, Yonghui Wang, Juan Wang, Li Liu

**Affiliations:** 1 School of Pharmacy, Fudan University, Shanghai, China; 2 Shanghai Drugability Biomass Product Evaluation Professional Public Service Platform, Center for Pharmacological Evaluation and Research, China State Institute of Pharmaceutical Industry, Shanghai, China; 3 Shanghai Children’s Medical Center affiliated to the Shanghai Jiao Tong University School of Medicine, Shanghai, China; 4 Department of Traditional Chinese Medicine, Xijing Hospital, The Fourth Military Medical University, Xi’an, Shaanxi 710032 China

**Keywords:** Skin stem cells, Stem-cell biotechnology, Drug screening

## Abstract

Skin infections caused by drug-resistant *Staphylococcus aureus* occur at high rates nationwide. Mouse primary epidermal organoids (mPEOs) possess stratified histological and morphological characteristics of epidermis and are highly similar to their derived tissue at the transcriptomic and proteomic levels. Herein, the susceptibility of mPEOs to methicillin-resistant S. aureus USA300 infection was investigated. The results show that mPEOs support USA300 colonization and invasion, exhibiting swollen epithelial squamous cells with nuclear necrosis and secreting inflammatory factors such as IL-1β. Meanwhile mPEOs beneficial to observe the process of USA300 colonization with increasing infection time, and USA300 induces mPEOs to undergo pyroptosis and autophagy. In addition, we performed a drug screen for the mPEO infection model and showed that vancomycin restores cell viability and inhibits bacterial internalization in a concentration-dependent manner. In conclusion, we establish an in vitro skin infection model that contributes to the examination of drug screening strategies and antimicrobial drug mechanisms.

## Introduction

Organoids are in vitro 3D cellular clusters derived exclusively from primary tissue, embryonic stem cells, or induced pluripotent stem cells that are capable of self-renewal and self-organization and exhibit organ functionality similar to that of the tissue of origin [1]. Organoid profile 3D structure, heterogeneity and the cellular function of major tissues are physiologically relevant in modeling human disease and predicting drug response, thus serving as a link between cell lines and in vivo models [2]. The skin is one of the largest organs in mammals. Its main functions include protection against fluid and electrolyte loss as well as physical, chemical and biological damage [3]. The construction of skin organoids has made tremendous progress in the past 5 years. Lee et al. utilized iPSC development to construct skin organoids that restore hair regeneration [4, 5]. Boonekamp et al. constructed epidermal organoids with highly self-organized structures by isolating adult stem cells [6]. Diao [7, 8] and Feldman et al. [9] constructed skin accessory gland-sweat gland organoids and sebaceous gland organoids, respectively.

Skin and soft-tissue infections are an important cause of morbidity and mortality among hospitalized patients and a major therapeutic challenge for clinicians. Early diagnosis, selection of appropriate antimicrobials and prompt surgical intervention are the keys to successful treatment [10]. Therefore, there is an urgent need to build an appropriate antimicrobial drug screening model. However, 2D epidermal cells do not encapsulate the in vivo cellular composition and physiological structure of the human epidermis, and the results of antimicrobial drug screening diverge from in vivo results. Three-dimensional skin equivalents [11–13] have a limited lifespan, a long modeling cycle and a low success rate, which may limit the large-scale application of this model system for drug testing. Organoids have proven to be a good model for studying infectious diseases and the mechanisms behind human-specific infectious agents [14]. Wang et al. [15] and Ma et al. [16] used skin organoids to simulate Trichophyton rubrum and SARS-CoV-2 infectious disease, respectively. Their research confirmed that skin organoids can be used as a novel system to model infectious skin diseases.

To date, the application of skin organoid infection models for drug testing has not been reported. In this study, we selected methicillin-resistant *Staphylococcus aureus* USA300, the most frequent cause of skin and soft-tissue infections [17, 18], and used mouse primary epidermal organoids (mPEOs) to model USA300 infections. We found that USA300 can colonize and internalize mPEOs, significantly reducing the cell viability of mPEOs. Furthermore, we demonstrated that USA300 furthered its infection process by inducing pyroptosis and autophagy. In addition, we identified the recovery of mPEO cell viability and changes in the number of bacterial internalized on mPEOs after antimicrobial drug administration. Based on these results, we propose a drug testing method suitable for the mPEO infection model, laying a theoretical foundation for its use as a preclinical antimicrobial drug screening model.

## Results

### Generation and characterization of mouse primary epidermal organoids (mPEOs)

**Fig. 1:**
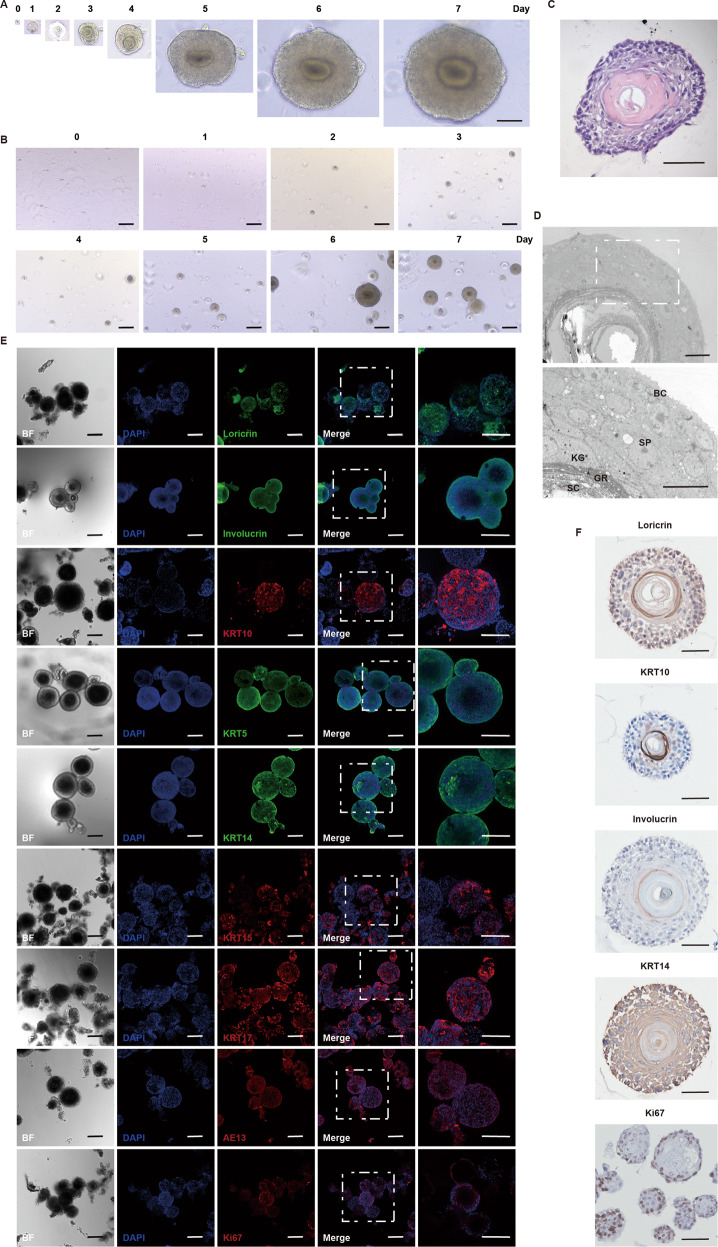
Characterization of mouse primary epidermal organoids (mPEOs). **A** Overview of mPEOs formed from single cells within 7 d (scale bars, 100 μm). **B** Increased number of mPEOs observed within 7 d in bright field (scale bars, 100 μm). **C** H&E staining of mPEOs cultured for 7 d (scale bars, 50 μm). **D** Ultrathin sections in TEM images of mPEOs (scale bars, 20 μm), SB stratum basale, SP stratum spinosum, GR stratum granulosum, KG keratohyalin granules, SC stratum corneum. **E** Immunofluorescence staining analysis of various skin-associated specific biomarkers in mPEOs (scale bars, 200 μm). **F** Immunohistochemical staining of paraffin-embedded mPEOs. Representative images of Loricrin, KRT10, Involucrin, KRT14 and Ki67 immunoreactivity in organoids (scale bars, 50 μm).

We collected skin tissue from newborn mice undergoing dissection and isolated epithelial cells through mechanical and enzymatic tissue disruption. Our method of constructing organoids is referred to as Boonekamp [6]. We embedded the isolated cells in Matrigel (Corning) at a density of 2500/10 µL and activated/blocked signaling pathways that are essential for the formation of mPEOs. Under optimized conditions, mPEOs formed within one week. The diameter of the organoids increased with increasing days, resulting in a 200–300 μm structure with a keratinizing inner core (Fig. 1A). The number of organoids also gradually increased (Fig. 1B).

Next, we analyzed the histological characteristics of mPEOs. The results of hematoxylin and eosin (H&E) staining indicated the morphology of mPEOs, with large flattened basal-like cells and sclerotized keratinized material in the center of organoid structures (Fig. 1C). The multilayered phenotype in organoids was further confirmed using transmission electron microscopy (TEM), with the structures including a stratum basale (SB): a layer of short, columnar basal cells; a stratum spinosum (SP): 4–10 layers of polygonal, larger spiny cells, with multiple oval, membrane-covered lamellated granules; a stratum granulosum (GR): 3–5 layers of flattened spindle-shaped cells, with more hyaline keratin granules (KG) in the cytoplasm; and a stratum corneum (SC): multiple layers of flattened keratinocytes, with fully keratinized cells and no nucleus or cytoplasm. SB is located on the outside of the organoids. SP, GR, and SC were found toward the center of the organoids (Fig. 1D).

The results of immunofluorescence staining showed that mPEOs expressed a variety of skin-specific biomarkers: a stratum granulosum marker, Loricrin, the stratum spinosum markers Involucrin and Cytokeratin-10 (KRT10^+^), the stratum basale markers Cytokeratin-5 (KRT5^+^), Cytokeratin-14 (KRT14^+^), and Cytokeratin-15 (KRT15^+^), the complex epithelial cell types markers Cytokeratin-17 (KRT17^+^) and Pancytokeratin (AE13^+^), and the proliferation-related marker Ki67^+^ (Fig. ^+^^+^^+^^+^^+^^+^^+^^+^^+^1E). We next performed immunohistochemical staining of mPEO-prepared paraffin sections. We found that the granulosum layer marker Loricrin and the spinous layer markers Involucrin and KRT10 were strongly immunoreactive in the center of the organoids. In addition, the organoids were positive for the stratum basale marker KRT14 and proliferation-related marker Ki67 (Fig. 1F). In conclusion, these data demonstrated that mPEOs, as mouse primary skin tissue-derived epidermal organoids, are well differentiated and possess a multilayered epidermal structure similar to that of mammalian skin.

### mPEO transcriptomes closely resemble gene expression signatures associated with the skin of origin

**Fig. 2:**
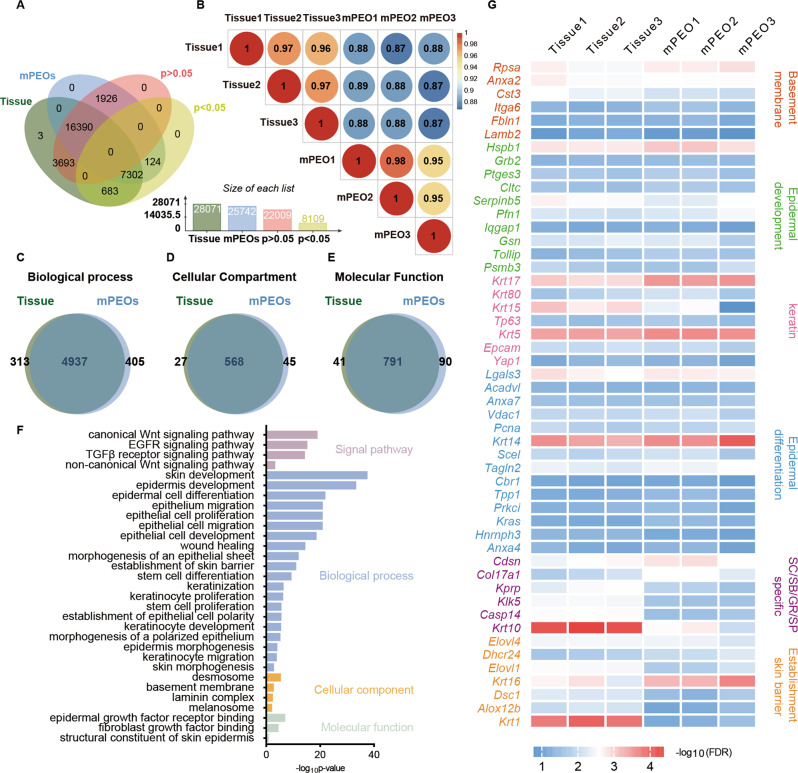
Comparisons of transcriptomes between mPEOs and their derived skin tissue. **A** Venn diagram [41] showing the overlapping genes between mPEOs and their derived skin tissue. **B** The Pearson correlation values of gene expression between mPEOs and tissues. Functional analysis of genes identified in mPEOs. Gene ontology (GO) using **C** biological process and **D** cellular compartment and **E** molecular function distribution. **F** GO analysis of genes identified in mPEOs. The top significantly enriched GO terms describing skin-related genes are shown with the -log of their *P* values. **G** Heatmap analysis of differences in the expression levels of genes involved in the basement membrane, epidermal development, keratin, epidermal differentiation, SC/SB/GR/SP specificity and establishment of the skin barrier in mPEOs and their derived skin tissues.

To assess the similarity of mPEOs to skin tissue, we compared their transcriptome data. Total RNA from mPEOs and their source skin tissue was extracted and sequenced by employing the polyA-enriched RNA-seq technique. Venn diagram overlapping regions showed 23,692 identical genes between mPEO and tissue. The number of genes that differed was 6429, of which 5622 were not significantly different (*p* > 0.05) (Fig. 2A). Based on 30121 genes that passed quality control, the Pearson correlation values for the three groups of mPEO and their derived tissues were 0.88, 0.88, and 0.87, respectively (Fig. 2B). Our GO enrichment analysis showed that the proportions of genes that were identical between organoids and tissues in terms of biological process, cellular compartment and molecular function were 4937/5655, 568/640, and 791/922, respectively (Fig. 2C–E). Biological process analysis showed that the genes identified in mPEOs were mainly enriched in skin developmental processes such as keratinocyte proliferation, epidermis development and epidermal cell differentiation, as well as skin physiological functions such as establishment of skin barriers, epithelial cell polarity, skin morphogenesis and wound healing. In addition, signaling pathways associated with skin development were identified, including canonical and noncanonical Wnt, EGFR and TGFβ receptors. Cellular compartment analysis revealed the presence of desmosomes, basement membranes and melanosomes in mPEOs (Fig. 2F). In addition, the expression of key marker genes involved in epidermal development, epidermal differentiation, skin barrier establishment and genes specific to the basement membrane, SC, SB, GR and SP were highly consistent between mPEOs and skin tissue (Fig. 2G).

### Comparison of proteomes demonstrates the similarity between mPEOs and their derived skin tissue

**Fig. 3:**
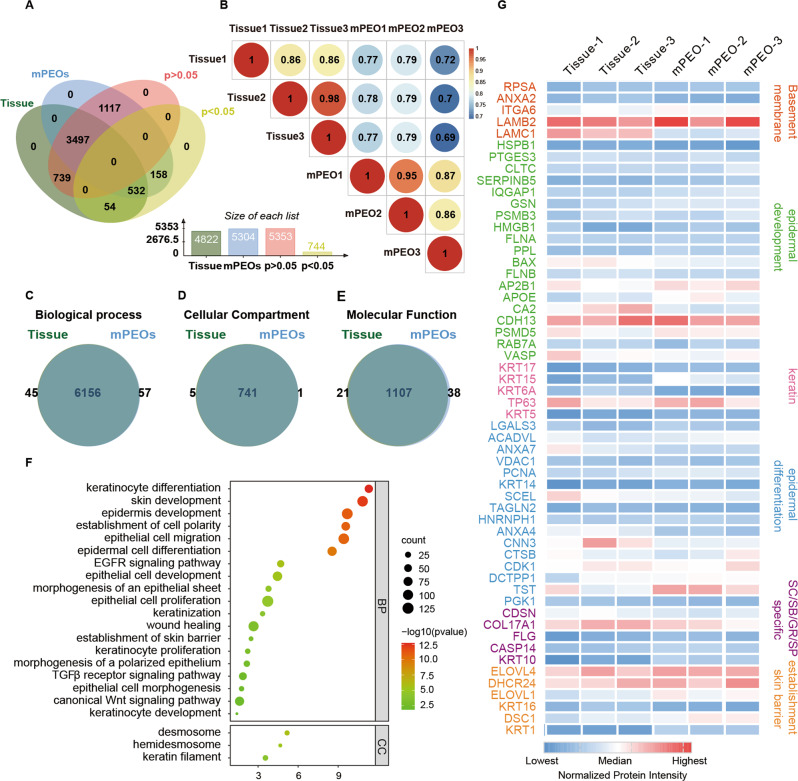
Comparisons of proteomes between mPEOs and their derived skin tissue. **A** Venn diagram showing commonly found proteins among mPEOs and their derived skin tissues. **B** The Pearson correlation values of protein expression between mPEOs and tissues. Venn diagram showing the overlapping proteins between mPEOs and their derived tissues are enriched in **C** biological process, **D** cellular compartment and **E** molecular function. **F** Biological process and cellular compartment analysis of the mPEO proteomic profile. **G** Heatmap analysis of differences in normalized intensity of proteins involved in basement membrane, epidermal development, keratin, epidermal differentiation, SC/SB/GR/SP specific and establishment skin barrier in mPEOs and their derived skin tissues.

To further indicate that mPEOs resemble and mimic skin tissue, we established proteomic profiles for mature mPEOs and their source tissues after culture was completed. Skin tissue and mPEOs contained 4822 and 5304 identified and quantified proteins, respectively. Among these, there were 5353 species with no significant difference (*p* > 0.05) and 744 species with a significant difference (*p* < 0.05) (Fig. 3A). Based on 6097 proteins that passed quality control, the Pearson correlation coefficients for the three groups of mPEO and their derived tissues were 0.77, 0.79, and 0.69, respectively (Fig. 3B). Our GO enrichment analysis showed that the proportions of proteins that were identical between organoids and tissues in terms of biological process, cellular compartment and molecular function were 6156/6258, 741/747, and 1107/1166, respectively (Fig. 3C–E). The proteins identified in mPEOs are involved in biological processes such as epithelial cell proliferation, epidermis development, epidermal cell differentiation, wound healing, and establishment of the skin barrier. Cellular compartment analysis revealed the inclusion of desmosomes, hemidesmosomes, and keratin filaments (Fig. 3F). We compared the normalized intensity of proteins involved in basement membrane, epidermal development, keratin, epidermal differentiation, SC/SB/GR/SP specific and establishment skin barrier among mPEO and its derived tissues, mostly without significant differences (Fig. 3G).

### USA300 infection and invasion in mPEOs

**Fig. 4:**
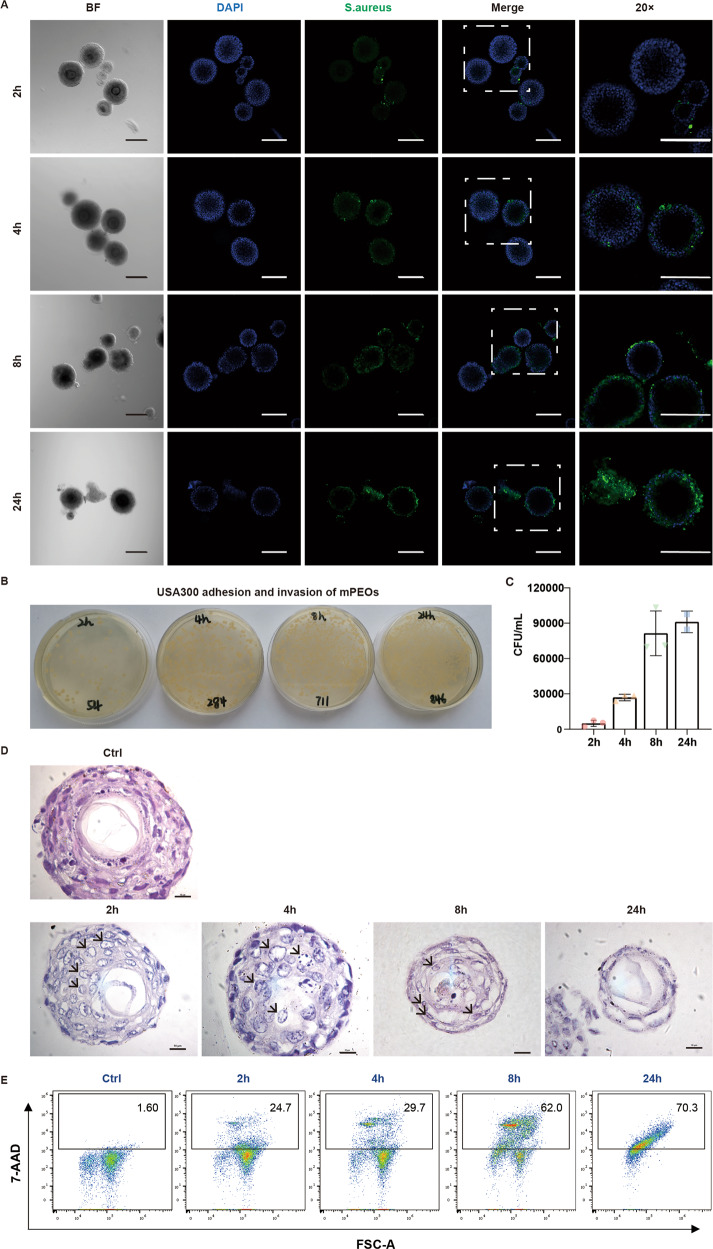
Modeling USA300 skin infection in mPEOs. **A** Immunofluorescence staining analysis of *Staphylococcus aureus*-specific biomarkers in mPEOs at 2, 4, 8, and 24 h of infection (scale bars, 200 μm). **B**, **C** CFU count of USA300 invading mPEOs at 2, 4, 8, and 24 h after gentamicin treatment for one hour. **D** H&E staining of infected mPEOs at 2, 4, 8, and 24 h (scale bars, 10 μm). **E** Flow cytometry 7-ADD staining results to assess the cell viability of mPEOs at 2, 4, 8, and 24 h of infection.

As seen above, we have established and characterized mPEOs (Fig. 1). We next assessed the feasibility of mPEOs in constructing models for skin diseases, especially infections. We selected USA300—a common, refractory methicillin-resistant *S. aureus* found in the American community that is commonly found in skin and soft-tissue infections. With S. aureus antibody immunofluorescence staining, we found that the number of USA300 colonies on mPEOs increased within 2, 4, 8, and 24 h (Fig. 4A). Through a gentamicin protection assay, we quantified the colony-forming unit (CFU) values of USA300-infected and invaded mPEOs. We found that at 2, 4, 8, and 24 h, with MOI = 10 infections, the numbers of adhesions and invasions on 1 × 10^5^ mPEOs were 4967 ± 2577, 26900 ± 2773, 81433 ± 18947, and 91100 ± 9192 CFU/mL, respectively (Fig. 4B, C). Compared to normal organoids, the infected mPEOs showed enlarged squamous epithelial nuclei and nuclear necrosis at 2 and 4 h. At 8 h, the mPEOs were vacuolated. At 24 h post infection, serious destruction of the mPEO structure occurred (Fig. 4D). We evaluated the viability of mPEOs using flow cytometry. At 2, 4, 8 and 24 h, the proportions of 7-ADD^+^ cells were 24.7%, 29.7%, 62.0% and 70.3%, respectively, indicating an increase in the number of cell deaths (Fig. 4E). Our data suggest that mPEOs possess the potential to model infectious diseases and that USA300 can colonize and invade organoids while reducing the cell viability of mPEOs.

### USA300 induces pyroptosis and autophagy to colonize mPEOs

**Fig. 5:**
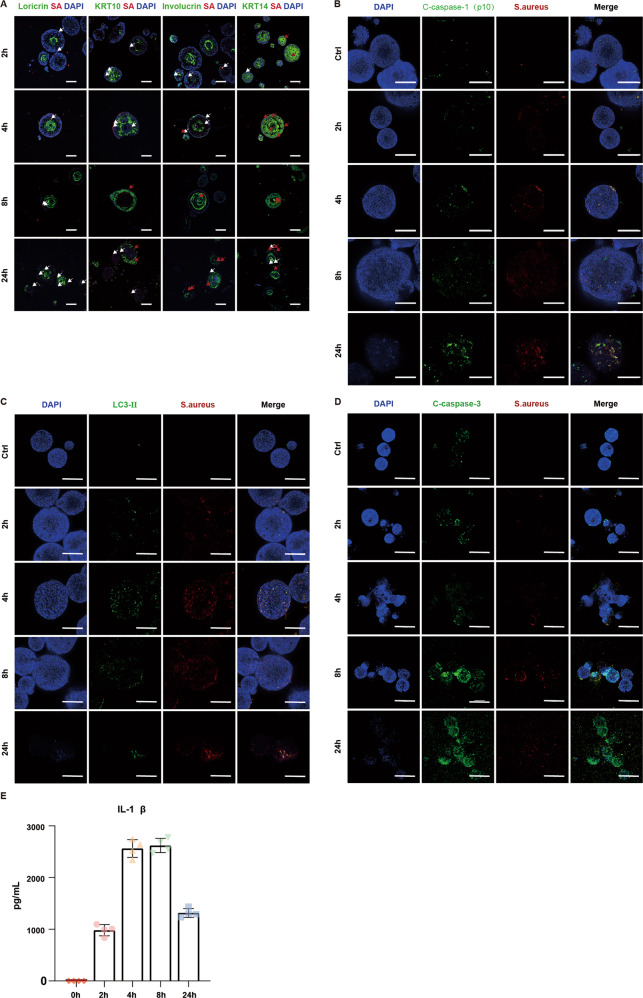
USA300 induces pyroptosis, autophagy and apoptosis in mPEOs. **A** Immunofluorescence staining analysis of *Staphylococcus aureus* biomarkers and Loricrin, KRT10, Involucrin, and KRT14 in mPEOs at 0, 2, 4, 8, and 24 h after USA300 infection. White arrows represent the location of USA300, and red arrows represent the colocalization of USA300 with Loricrin, KRT10, Involucrin or KRT14. (Scale bars, 200 μm). **B** Immunofluorescence staining analysis of the *Staphylococcus aureus* biomarker and Cleaved-caspase-1 (p10) in mPEOs 0, 2, 4, 8, and 24 h after USA300 infection (scale bars, 200 μm). **C** Immunofluorescence staining analysis of *Staphylococcus aureus* biomarkers and LC3-II in mPEOs after USA300 infection at 0, 2, 4, 8, and 24 h (scale bars, 200 μm). **D** Immunofluorescence staining analysis of *Staphylococcus aureus* biomarkers and Cleaved-caspase-3 in mPEOs after USA300 infection at 0, 2, 4, 8, and 24 h (scale bars, 200 μm). **E** IL-1β production was determined using ELISA at 0, 2, 4, 8, and 24 h after mPEOs were infected with USA300.

We have demonstrated that USA300 can achieve colonization of mPEOs and reduce their cell viability (Fig. 4). We further investigated the colonization process of USA300 on mPEOs. By colabeling with antibodies against S. aureus and skin stratification biomarkers, we found that S. aureus mostly colonized the periphery of the organoids and colocalized with the stratum basal marker KRT14 at 2 and 4 h; at 8 and 24 h, the peripheral cells of the organoids died, as evidenced by attenuation of the DAPI signal. S. aureus invaded the interior of the organoids and colocalized with KRT14 and the stratum spinosum markers Involucrin and KRT10 (Fig. 5A).

Confocal imaging of infected mPEOs was performed to semiquantitatively assess the accumulation of biomarkers for pyroptosis, autophagy and apoptosis. A cardinal feature of pyroptosis is the requirement for caspase-1 activation. Cleaved-caspase-1, which includes the p10 and p20 subunits, is a hallmark of caspase-1 activation. With increasing duration of infection, positive signals for the p10 subunit were detected, and we found that on mPEOs, it colocalized in regions colonized by USA300 (Fig. 5B). LC3-II, a marker for autophagosome formation, was detected in infected mEPOs and colocalized with USA300 (Fig. 5C). Cleaved-caspase-3, a marker for apoptosis, was also detected on infected mPEOs, but mostly not colocalized with USA300 (Fig. 5D). In conclusion, during infection, USA300 induced pyroptosis and autophagy at sites of mPEOs colonization and invasion, and apoptosis at other locations.

Induction of autophagy leads to the consumption of nonessential cellular components. Autophagic degradation limits pyroptosis and IL-1β production [19], which protects USA300, avoiding keratinocyte-mediated clearance. We collected supernatants of mPEOs at 0, 2, 4, 8, and 24 h after USA300 infection and measured IL-1β expression levels of 0 ± 0, 982.00 ± 108.55, 2562.62 ± 172.03, 2621.46 ± 137.35 and 1315.83 ± 86.14 pg/mL respectively by ELISA (Fig. 5E). We found that compared with the control group, IL-1β was significantly expressed at 2, 4 and 8 h, with the expression decreasing at 24 h. We speculate that mPEOs release inflammatory factors, including IL-1β, through pyroptosis to clear the USA300. USA300, in turn, may also escape keratinocyte-mediated clearance by inducing autophagy of mPEOs, promoting degradation of inflammasomes, and achieving colonization.

### Evaluation of drug testing methods in mPEO infection models

**Fig. 6:**
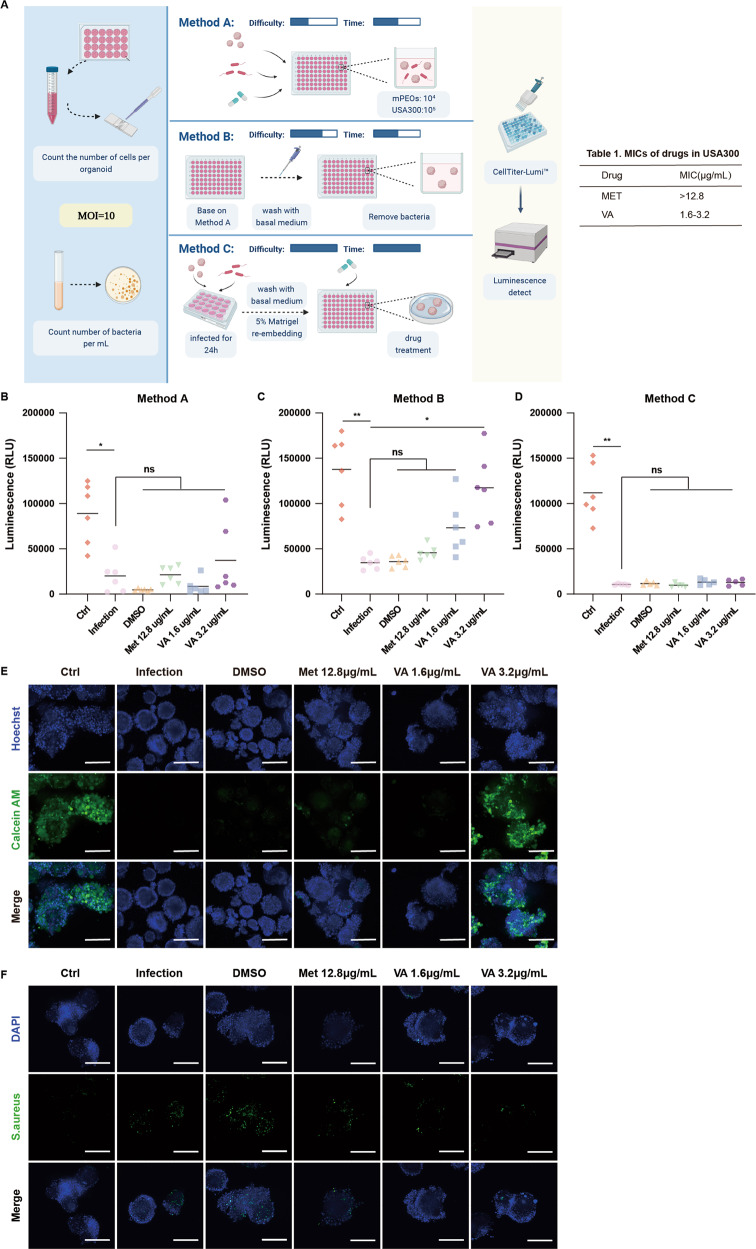
Drug testing methods for mPEOs in the USA300 infection model. **A** Overview of the three methods protocol steps (created with BioRender.com). The viability of infected mPEOs after treatment with DMSO, 12.8 μg/mL methicillin, 1.6 μg/mL and 3.2 μg/mL vancomycin was analyzed using a CellTiter-Lumi™ ATP assay. **B** Method A (*n* = 6, Ctrl group: 89119.28 ± 33928.97; Infection group: 20019.28 ± 18394.53; DMSO group: 4806.33 ± 1301.42; Met 12.8 μg/mL group: 21418.17 ± 9640.48; VA 1.6 μg/mL group: 8560.96 ± 8868.99; VA 3.2 μg/mL group: 37237.76 ± 39975.97). **C** method B (*n* = 6, Ctrl group: 137746.08 ± 39574.03; Infection group: 34833.32 ± 7176.01; DMSO group: 35910.03 ± 6193.07; Met 12.8 μg/mL group: 45786.43 ± 7628.59; VA 1.6 μg/mL group: 73176.32 ± 31063.43; VA 3.2 μg/mL group: 117408.70 ± 38819.92). **D** method C (*n* = 5, Ctrl group: 111847.12 ± 31074.82; Infection group: 10614.81 ± 633.50; DMSO group: 11657.73 ± 2164.49; Met 12.8 μg/mL group: 9753.12 ± 2082.89; VA 1.6 μg/mL group: 13274.31 ± 3204.52; VA 3.2 μg/mL group: 12869.72 ± 3419.86). **E** Fluorescent staining of mPEOs labeled with Calcein AM and Hoechst after treatment with DMSO, 12.8 μg/mL methicillin, 1.6 μg/mL and 3.2 μg/mL vancomycin (scale bars, 200 μm). **F** Immunofluorescence staining analysis of *Staphylococcus aureus*-specific biomarkers in infected mPEOs after treatment with DMSO, 12.8 μg/mL methicillin, 1.6 μg/mL or 3.2 μg/mL vancomycin (scale bars, 200 μm).

Next, we applied mPEOs to drug testing. mPEO infection followed by drug testing amounts to an added process of disease modeling. We devised three methods to evaluate the most accurate and simplest drug testing assay for infected mPEOs. Briefly, method A: tryple-released mPEOs from Matrigel, MOI = 10 were coincubated with USA300 and treated with the indicated drug concentrations for 24 h, and mPEO viability was determined using the CellTiter-Lumi™ luminescence cell viability assay kit. Method B: based on method A, the supernatant was discarded at the end of the drug treatment and washed with basal medium before performing the CellTiter-Lumi™ viability assay to exclude the effect of bacterial lysis on the accuracy of the kit. Method C: mPEOs were coincubated with USA300 for 24 h, and the bacteria were washed off. The CellTiter-Lumi™ viability assay was performed after re-embedding with 5% Matrigel and treatment with the indicated drug concentrations for 24 h (Fig. 6A).

We selected clinically used antibiotics methicillin and vancomycin for drug testing. We measured the minimum inhibitory concentrations (MICs) of methicillin and vancomycin to be >12.8 μg/mL and 1.6–3.2 μg/mL, respectively (Table 1). We treated infected mPEOs with 12.8 μg/mL methicillin, 1.6 and 3.2 μg/mL vancomycin. The viability levels of mPEOs after drug treatment were examined according to the three methods described above. First, methods A, B and C detected mPEOs in the control group, and the values were stable and homogeneous and largely conformed to the kit criteria (the number of 10^4^ cells corresponds to a chemiluminescence reading on the order of 10^5^-10^6^). Method B showed that methicillin did not reverse cell death caused by infection, while vancomycin restored cell viability in a concentration-dependent manner. Method A and method C showed no significant difference between the drug-treatment groups and the infection group, and we considered them less accurate (Fig. 6B–D). The time required for methods A and B was 24 h, while method C required 48 h. The order of both experimental difficulty and cost was C > B > A. In terms of the overall evaluation, we considered method B to be the most suitable method for drug sensitivity assays after modeling mPEO infection.

**Table 1:** MICs of drugs in USA300.

Drugs	MIC(μg/mL)
Methicillin	>12.8
Vancomycin	1.6–3.2

Following method B at the end of drug treatment, we visualized mPEOs using Hoechst and labeled live cells of mPEOs with Calcein AM dye. We found that the fluorescence signal of Calcein AM in mPEOs almost completely disappeared after infection, indicating a significant decrease in cell viability. Calcein AM showed positive expression when mPEOs were treated with 3.2 μg/mL vancomycin, indicating that vancomycin could restore the cell viability of mPEOs (Fig. 6E). We assessed the degree of internalization of USA300 using immunofluorescence techniques to label antibodies against S. aureus and laser confocal in the Z-axis to scan mPEOs at 5 μm/layer, superimposing the maximum fluorescence signal of each layer. Methicillin did not affect USA300 internalization in mPEOs, and vancomycin reduced USA300 internalization in a concentration-dependent manner (Fig. 6F).

## Discussion

Bacterial skin infections are a major societal health burden and are increasingly difficult to treat owing to the emergence of antibiotic-resistant strains such as community-acquired methicillin-resistant *S. aureus* [20]. The scientific community has established several in vitro models for skin infections that help us understand the pathophysiology of the disease and develop therapeutic strategies, represented by classic reconstructed epidermis using the ALI method. In this study, we successfully constructed mouse primary epidermal organoids (mPEOs) with multilayer structures and developed a novel epidermal model for USA300 infection based on this. Importantly, mPEOs amplify at a faster rate, supporting cryopreservation and resuscitation. Our results show that mPEOs have been passed for 11 generations (Fig. S1), and our disease modeling can be completed within 9 days. Therefore, mPEOs are more suitable for large-scale and rapid applications in industrial or clinical projects than reconstructed epidermis. Based on our RNA sequencing and protein mass spectrometry results, mPEOs are highly similar to the source tissues at the transcriptional and proteomic levels. Since organoids can serve as a versatile tool allowing targeted gene editing, drug testing, and transcriptome analysis [21], mPEOs can provide a platform for basic and translational research focused on host-microbe interactions.

We observed that when cocultured with mPEOs, USA300 can directly enter the outer “basal” keratinocytes and avoid the step of invasion into the stratum corneum. We suggest that this is more similar to the epidermal wound injury infection process. The main reason for this process is the structure of mPEOs, where the basal layer is located on the outside of the organoid while the spinous layer, granular layer and stratum corneum are located in the center of the organoid. The receptor for the ECM protein β1 integrin is extremely important in the process of epithelial organoid polarity reversal [22]. This may also be a major factor in the “basal-out” behavior of epithelial organoids such as mPEOs. We have tried microinjection techniques used in other studies [23, 24], but mPEOs are noncystic organoids with limited central space, making them difficult and time-consuming to manipulate. Subsequently, we will try to use the protocol published by Co et al. treating the organoid with EDTA and then suspending its culture, based on the principle that EDTA chelation of cations and disruption of the interaction of the ECM with the basolateral β-1 integrin receptor will trigger coordinated epithelial movement and morphological rearrangement, allowing the polarity of the organoid to ectopically flip [25]. This allows the cuticle of mPEOs to come into direct contact with microorganisms. further facilitating the study of host‒pathogen interactions with the skin epithelium.

Our cultured mPEOs contain intact epidermal structures and lack dermis, accessory glands, and immune cells, resulting in a model that does not accurately summarize the response of infection to all skin components. Among in vitro skin models, 3D skin equivalents derived from the ALI approach reproduce the function of the dermis and immune cells by coculture with fibroblasts and immune cells (e.g., Langerhans cells, centrophages, macrophages, and different types of lymphocytes). However, the problem of regeneration of accessory glands has not been solved. The ALI model is not suitable for high-throughput drug screening and sequencing analysis for antimicrobial target exploration. Many current studies focus on organoid systems cocultured with immune cells [26]. Subsequently, we will investigate the best way to introduce the immune system. One way is to add immune cells to mPEOs after they are cultured, as in Norman [27] and Loryn [28], to determine the composition of the culture medium and the ratio of immune cells to achieve long-term coexistence. Another way is to construct organoids with primary tissue microdissection rather than single cells to preserve multiple endogenous immune cell types, as in Li et al. [29, 30]. Furthermore, Lee et al. [4] utilized iPSC-derived skin organoids to differentiate relatively complete skin components, including epidermis, dermis, hair follicles, sebaceous glands, nerves and adipocytes. However, their need for a 90–150 day culture cycle limits their application in high-throughput drug testing.

There are few articles on the establishment of systems for skin organoid infection, two of which use human-derived cells. Ma et al. [16] used hiPSC-derived skin organoids to demonstrate that KRT17^+^ hair follicles can be infected by SARS-CoV-2 and are associated with impaired development of hair follicles and epidermis. The potential pathological mechanism between COVID-19 and hair loss was explored. Wang et al. [15] used epidermal stem/progenitor cells-derived human primary epidermal organoids to mimic *Trichophyton Rubrum* (*T. rubrum*) infection, and their study showed that the inhibition of IL-1 signaling may be the pathogenic mechanism of chronic and recurrent infections with the slight inflammation caused by *T. rubrum* in human skin. These two studies confirm the feasibility of skin organoids for infection modeling, which as an in vitro bionic model, separate from the complex systems in vivo, are more advantageous in elucidating key pathological phenotypes and mechanisms.

Drug testing protocols for tumor organoids are relatively well established [31–33], whereas drug-sensitive testing of normal-like organs after infection has been less reported [34–37]. Drug testing after organoid infection corresponds to an added process in disease modeling. Currently, a common drug testing protocol for tumor organoids is performed using the CellTiter-Glo® Kit (Progema, G7570), an ATP-based homogeneous method for quantifying the number of viable cells in culture. However, organoid infection models can introduce microorganisms and ATP released by microbes can interfere with test results. Our data also confirm that the sensitivity of the kit can be improved by removing the bacteria from the culture medium. No suitable removal method has been found for the effect of bacteria internalized in the organoid. We also tried to perform the drug testing in a “Matrigel re-embedding” manner for tumor organoids (Fig. 6A-Method C), but for the mPEO infection model we found that this approach further reduced the cell viability of mPEOs after infection and failed to restore the cell viability of mPEOs after drug treatment.

In conclusion, our study demonstrates that mPEOs can serve as a new platform for ex vivo studies, disease modeling, drug screening, and target discovery. We have established suitable disease modeling methods for mPEOs as well as drug sensitivity assays. Our study confirms that mPEOs can be expected to complement the existing library of infection models and provide guidance for drug testing of other organoid infection models.

## Methods and materials

### Murine epidermal keratinocyte isolation and mPEO culture

We collected telogen back skin from C57BL/6 mice. The tissues were digested with 2.5 U/mL dispase (Stemcell) for 1 h at 37 °C. The epidermis was carefully peeled off with tweezers and cut into small pieces. The minced tissue pieces were subsequently digested with 0.25% Trypsin (Gibco) for 20–30 min. After filtering through a 70 μm nylon cell strainer and centrifugation, we finally obtained single primary epidermal cells. Keratinocytes were plated at a density of 2500 cells per 10 μL Matrigel. Cells were cultured in medium consisting of advanced DMEM/F12 (Gibco) supplemented with penicillin/streptomycin (100 U/L; Gibco), HEPES (10 mM; Gibco), GlutaMAX (1×; Gibco), B27 supplement (50× stock; Gibco), N-acetylcysteine-1 (1 mM; Sigma‒Aldrich), bovine serum albumin (0.1%; Sigma–Aldrich), forskolin (10 μM; BioGems), EGF (50 ng/mL; Peprotech), Wnt3a (100 ng/mL; Peprotech), Y-27632 (10 μM; BioGems) and A83-01 (1 μM; Sellect) that was added after 2 days of culture.

mPEOs could be removed from Matrigel by incubating with 1 U/mL Dispase for 60 min at 37 °C and further dissociated into small clumps of cells or single cells using Tryple (Gibco). mPEOs were passaged at a 1:3–1:4 ratio every 7 days. mPEOs could be dissociated into small clumps or single cells, cryopreserved in serum-free cryopreservation medium (StemCell), placed in −80 °C or liquid nitrogen, and recovered with the optimized organoid medium.

### Bacterial strain culture

*S. aureus* strain USA300, a methicillin-resistant strain derived from adolescent patients with severe sepsis syndrome at Texas Children’s Hospital, was purchased from ATCC (BAA-1717). USA300 were cultured in tryptic soy broth (TSB) for 3 h to log-phase with shaking at 220 rpm at 37 °C.

### Hematoxylin & Eosin (H&E) and Immunohistochemistry staining

mPEOs could be removed from Matrigel by incubating with 1 U/mL Dispase for 60 min at 37 °C. Organoids were centrifuged at 300 × *g* for 5 min. The medium was aspirated and the organoids were resuspended in 4% paraformaldehyde (PFA) and incubated for 1 h at 37 °C. The fixed mPEOs were then embedded in 2% agarose. Subsequently, organoids were dehydrated and embedded in paraffin wax. Hematoxylin & eosin (H&E) and immunohistochemical staining were performed on 4-µm sections of paraffin-embedded organoids. Paraffin sections of organoids were dewaxed, and antigen retrieval was performed by either pretreatment with EDTA Antigen Retrieval Solution pH 9 (50×, Beyotime) or boiling in citrate buffer pH 6 (50×, Beyotime). Subsequently, slides were incubated in blocking buffer consisting of 0.1% Triton X-100 (Beyotime) and 3% bovine serum albumin (BSA; Sigma‒Aldrich) in PBS for 1 h at room temperature. Slides were incubated overnight at 4 °C with the following primary antibodies: rabbit anti-Involucrin (AF0186, 1:500, Affbiotech), rabbit Loricrin (ab85679, 1:200, Abcam), rabbit anti-KRT10 (ab76318, 1:400, Abcam), and mouse anti-KRT14 (abs131470-50 µg, 1:500, absin). After washing, the slides were incubated with an SABC-HRP Kit with anti-rabbit IgG (1:50, P0615, Beyotime) for an hour at room temperature, after which the slides were washed and developed using DAB (3,3′-diaminobenzidine tetrahydrochloride hydrate, P0203, Beyotime) development. Finally, neutral resin was used to seal the film.

### Immunofluorescence staining

Following Hans Clevers [38], mPEOs were released from the Matrigel and incubated with cell recovery solution reagent (Corning) on a horizontal shaker at 4 °C (60 rpm) for 30–60 min. The pellet of organoids was gently resuspended in 1 ml of PFA and incubated at 4 °C for 45 min. Then, cold OWB (Organoid Washing Buffer, including 0.1% Triton X-100 + 0.2% BSA) was used to transfer the appropriate amounts of organoids per staining to a low-adherence/suspension 24-well plate and incubated at 4 °C for 15 min. Then, 200 µL of OWB with primary antibodies (2× concentration) was added to each well and incubated overnight at 4 °C with mild rocking/shaking (60 rpm on a horizontal shaker). The cells were incubated with 200 µL of OWB-diluted secondary antibody (2× concentration) for 1 h at room temperature in the dark. The OWB was removed and incubated with DAPI for 1 h at room temperature in the dark. The images were captured using a Nikon ECLIPSE Ti2. A complete list of the primary and secondary antibodies used is provided in Table 1.

### Transmission electron microscopy

The mPEOs released from Matrigel were then fixed with 2.5% glutaraldehyde at 4 °C overnight. Centrifuged at 300 × *g* for 5 min to remove the supernatant. Add 0.1 M phosphate buffer PB (pH 7.4) and rinse for 3 min before centrifugation and repeat washing 3 times. Then wrapped in the 1 % agarose solution. Agarose blocks with mPEOs were fixed with 1% OsO4 in 0.1 M PB (pH 7.4) for 2 h at room temperature in dark. After removing OsO4, the Agarose blocks are rinsed in 0.1 M PB (pH 7.4) for 3 times, 15 min each. Place on 30%, 50%, 70%, 80%, 95%, 100%, 100% alcohol for dehydration for 20 min each time and 100% acetone twice for 15 min each time. Then resin penetration and embedding as followed: Acetone: EMBed 812 = 1:1for 2–4 h at 37 °C; Acetone: EMBed 812 = 1:2 overnight at 37 °C; pure EMBed 812 for 5–8 h at 37 °C. Pour the pure EMBed 812 into the embedding models and insert the agarose blocks into the pure EMBed 812, and then keep in 37 °C oven overnight. The embedding models with resin and mPEOs were moved into 65 °C oven to polymerize for more than 48 h. And then the resin blocks were taken out from the embedding models and were cut to 60–80 nm thin on the ultramicrotome, then fished out onto the 150 meshes cuprum grids with formvar film. Two percent uranium acetate saturated alcohol solution avoid light staining for 8 min, rinsed in 70% ethanol for 3 times and then rinsed in ultra-pure water for 3 times. 2.6% Lead citrate avoid CO2 staining for 8 min, and then rinsed with ultra-pure water for 3 times. After drying with the filer paper, the cuprum grids were put into the grids board and dried overnight at room temperature. The stain sections were examined with a Hitachi HT7800 Series 120 kV transmission electron microscope (TEM).

### RNA sequencing

According to the results of Wang et al. [39], we released mPEOs cultured for 7 days from Matrigel using 1 U/mL Dispase at 37 °C for 1 h, and the derived tissue was stored in RNAlater (Thermo Fisher, AM7020). The total RNA of the mPEOs and tissues was extracted using TRIzol according to the manufacturer’s instructions. One microgram of total RNA was used for subsequent library preparation. Poly(A) mRNA isolation was performed using Oligo(dT) beads. mRNA fragmentation was performed using divalent cations and high temperatures. Priming was performed using Random Primers. First-strand cDNA and second-strand cDNA were synthesized. The purified double-stranded cDNA was then treated to repair both ends, and dA-tailing was added in one reaction, followed by T-A ligation to add adapters to both ends. Size selection of adapter-ligated DNA was then performed using DNA clean beads. Each sample was then amplified with PCR using P5 and P7 primers, and the PCR products were validated. Then, libraries with different indices were multiplexed and loaded on an Illumina HiSeq/Illumina NovaSeq/MGI2000 instrument for sequencing using a 2 × 150 paired-end (PE) configuration according to the manufacturer’s instructions. Original data was uploaded to the Sequence Read Archive (accession number: SRR22497643, SRR22497644, SRR22497645, SRR22497646, SRR22497647, SRR22497648).

### Proteomic analysis method

#### Protein extraction and concentration determination

Following Wang et al. [39]. The supernatant of mPEOs was removed and washed twice with PBS; incubated with 1U/mL Dispase (Advanced DMEM/F12 dilution) at 37 °C for 30 min; centrifuged at 300 × *g* for 5 min to remove the supernatant; repeated twice to exclude interference from Matrigel. mPEOs and tissues were collected, and an appropriate amount of RIPA lysis buffer (Biotechwell, WB0101) and protease inhibitors (Biotechwell, WB0122) was added and shaken thoroughly on a shaker, followed by lysis on ice for 1 h. The supernatant was collected by centrifugation at 13300 rpm for 15 min. The protein concentration was determined according to the standard protocol of the BCA protein assay kit (Biotechwell, WB0123).

#### Protein tryptic digestion

Proteins were reduced in 5 mM dithiothreitol at 56 °C for 30 min and then alkylated in 15 mM iodoacetamide at room temperature for 30 min in darkness. The reaction was quenched with 30 mM cysteine at room temperature for an additional 30 min. Protein samples underwent trypsin digestion (enzyme-to-substrate ratio of 1:50 at 37 °C for 16 h) followed by desalting through MonoSpin C18 cartridges and vacuum-drying by Speed Vac. The peptide residues were reconstituted in water containing 0.1% formic acid and centrifuged at 14,000 rpm for 10 min prior to nano-LC‒MS/MS analysis.

#### Nano-LC‒MS/MS

Peptide samples were analyzed on a nano-HPLC (nanoElute, Bruker Daltonics) onto 250 mm × 75 μm ID pulled emitter columns (IonOptiks) packed with 1.6 μm C18-particles and heated at 50 °C in a column oven. The mobile phases consisted of 0.1% (v/v) formic acid (FA) in water (phase A) and acetonitrile (phase B). Samples were separated by a 60 min stepped gradient ranging from 2 to 30% B at a flow rate of 400 nL/min. Peptides were detected on a timsTOF Pro instrument (Bruker Daltonics) operated in PASEF mode. TIMS accumulation times were fixed at 100 ms, while the ion mobility separation was fixed to 100 ms. The range of mobility values was 0.45–1.45 vs./cm^2^ (1/K0), and the covered m/z range was 100–1700 *m*/*z*.

#### MS database searching

MS raw files generated by LC‒MS/MS were searched against the UniProt mouse proteome database (version 2021-01-01) using PaSER (version 1.0) software. The protease was trypsin. Up to 2 missed cleavages were allowed. Carbamidomethyl (C) was considered a fixed modification. The variable modifications were oxidation (M) and acetylation (protein N-term). The cutoff of the false discovery rate (FDR) using a target-decoy strategy was 0.01 for both proteins and peptides.

### USA300 infection of mPEOs

Before infection, mPEOs were released from Matrigel by incubating with 1 U/mL Dispase for 1 h at 37 °C. Released mPEOs were suspended in the culture medium and infected with USA300 at a multiplicity of infection (MOI) of 10 for 2, 4, 8, and 24 h at 37 °C in 5% CO_2_. Specifically, we collected the mPEO suspension and mixed it with a Pasteur pipette. A total of 1 mL was centrifuged at 300 × *g* for 5 min, and the supernatant was aspirated. Organoids were resuspended in 2 mL TrypLE (Gibco) and incubated for 15 min at 37 °C. Subsequently, organoids were disrupted by pipetting up and down at least 10 times until the mPEOs were disrupted to single cells, accurately counting cells to estimate mPEOs. Then, 10^5^ mPEOs and 10^6^ CFU/mL USA300 were resuspended in 1 mL of Advanced DMEM/F12 per well in a 24-well plate. After infection, the mPEOs were washed twice with the basal medium for subsequent CFU count, drug testing or immunofluorescence analysis.

### Gentamicin protection assay

After the infection time arrived, extracellular bacteria were killed by the addition of gentamicin (MCE, HY-A0276) to a final concentration of 500 μg/mL for 1 h at 37 °C. To quantify intracellular bacteria, mPEOs were disassociated with Tryple at 37 °C for 15 min, pipetting up and down more than 10 times, and dilutions were plated on LB agar plates for CFU enumeration.

### Flow cytometry

Following Hans Clevers [40], after infection, mPEOs were harvested from the culture in 1 mL of ice-cold (4 °C) DMEM and centrifuged for 5 min at 300 × *g*, and the supernatant was discarded. Then, mPEOs were resuspended in TrypLE (Gibco) and incubated for 15 min at 37 °C. Subsequently, organoids were disrupted by pipetting up and down at least 10 times until the mPEOs were dissociated into single cells. The cells were resuspended in 100 μL PBS with 5 μL 7-ADD (BioGems, 61410-00-200) and incubated at room temperature for 15 min in the dark. Filter with a 40 μm cell strainer. The cells were run on a Beckman CytoFlex, and the ratio of 7-ADD-positive cells to total cells was assessed to establish mPEO viability.

### Detection of IL-Iβ levels by ELISA

At the time point of infection for 2, 4, 8, and 24 h, mPEO culture supernatants were collected for IL-1β enzyme-linked immunosorbent assay (ELISA) (MULTI SCIENCES, EK201B/3-48). The operation followed the standard experimental protocol of the ELISA kit.

### Minimum inhibitory concentration (MIC) assay

The MIC was determined using the microdilution method according to Clinical and Laboratory Standards Institute (CLSI) guidelines. USA300 was diluted in Mueller–Hinton broth (MHB) and introduced into 96-well plates. After that, methicillin (MCE, HY-B0974) and vancomycin (Sigma‒Aldrich, V2002-100MG) prepared in 10 mM stock solutions were subjected to twofold serial dilution in a 96-well plate to final concentrations of 1280 to 1.25 μg/mL, in triplicate. The MIC was defined as the lowest drug concentration that completely inhibited visible bacterial growth.

### Drug testing for USA300-infected mPEOs

#### Method A

The cell number assessment of mPEOs was the same as “USA300 infection to mPEOs”. In black 96-well plates, each well contained 100 µL Advanced DMEM/F12 resuspended 10^4^ mPEOs, 10^5^ USA300 and the indicated concentrations of drug (methicillin: 12.8 μg/mL; vancomycin: 1.6, 3.2 μg/mL) or DMSO control. After drug treatment for 24 h, 100 µL of CellTiter-Lumi™ Luminescent Cell Viability Assay Kit (CTL, Beyotime, C0065S) was added to each well and incubated at room temperature for 1 h in the dark, and luminescence parameters were detected on a microplate reader to evaluate the activity of mPEOs.

#### Method B

USA300 infection and drug treatment were the same as in Method A. The difference was that after drug treatment for 24 h, 80 µL of supernatant was removed (to avoid the loss of mPEOs) and supplemented with Advanced DMEM/F12 to a total volume of 100 µL, and mPEO viability assays were performed using CTL.

#### Method C

mPEOs were harvested from Matrigel and infected with USA300 at MOI = 10 for 24 h. Then, the mPEOs were washed twice with basal medium. Infected mPEOs were suspended with 5% Matrigel and dispensed onto 96-well plates as 20 μL droplets and overlaid with 100 μL of medium per well. Each well contained up to 10,000 cells. USA300-infected mPEOs were treated with DMSO, 12.8 μg/mL methicillin, or 1.6 and 3.2 μg/mL vancomycin immediately for 24 h after re-embedding. Finally, mPEO viability assays were performed using CTL.

### LIVE cell immunofluorescence staining

At the end of drug treatment, mPEOs were washed once with PBS and stained for 2 h with a mixture of 2.5 µM calcein AM (eBioscience, 65-0853-39) and 1× Hoechst (Beyotime, C1028), and fluorescent imaging was performed.

### Quantification and statistical analyses

All data are presented as the mean of at least two independent experiments with corresponding error bars of standard deviation (SD) or the standard error of the mean (SEM). Data analysis was performed using GraphPad Prism version 8.02 using one-way or two-way ANOVA with post hoc multiple comparison tests or Student’s *t* test, as specified in the figure legends. Statistical significance is indicated with *p < 0.05, **p < 0.01 and ***p < 0.001.

## Supplementary information

Reproducibility Checklist

Supplementary Figure 1

Supplementary Table 1

Supplementary Table 2

Supplementary Table 3

## Data Availability

All datasets generated and analyzed during this study are included in this published article and its Supplementary Information files. Additional data are available from the corresponding author on reasonable request.

## References

[1] Fatehullah A, Tan SH, Barker N (2016). Organoids as an in vitro model of human development and disease. Nat Cell Biol.

[2] Jensen C, Teng Y (2020). Is it time to start transitioning from 2D to 3D cell culture?. Front Mol Biosci.

[3] Blanpain C, Fuchs E (2009). Epidermal homeostasis: a balancing act of stem cells in the skin. Nat Rev Mol Cell Biol.

[4] Lee J, Rabbani CC, Gao H, Steinhart MR, Woodruff BM, Pflum ZE (2020). Hair-bearing human skin generated entirely from pluripotent stem cells. Nature.

[5] Lee J, Bӧscke R, Tang P-C, Hartman BH, Heller S, Koehler KR (2018). Hair follicle development in mouse pluripotent stem cell-derived skin organoids. Cell Rep.

[6] Boonekamp KE, Kretzschmar K, Wiener DJ, Asra P, Derakhshan S, Puschhof J (2019). Long-term expansion and differentiation of adult murine epidermal stem cells in 3D organoid cultures. Proc Natl Acad Sci USA.

[7] Diao J, Liu J, Wang S, Chang M, Wang X, Guo B (2019). Sweat gland organoids contribute to cutaneous wound healing and sweat gland regeneration. Cell Death Dis.

[8] Sun X, Xiang J, Chen R, Geng Z, Wang L, Liu Y (2021). Sweat gland organoids originating from reprogrammed epidermal keratinocytes functionally recapitulated damaged skin. Adv Sci.

[9] Feldman A, Mukha D, Maor II, Sedov E, Koren E, Yosefzon Y (2019). Blimp1(+) cells generate functional mouse sebaceous gland organoids in vitro. Nat Commun.

[10] Cardona AF, Wilson SE (2015). Skin and soft-tissue infections: a critical review and the role of telavancin in their treatment. Clin Infect Dis Publ Infect Dis Soc Am.

[11] Poumay Y, Dupont F, Marcoux S, Leclercq-Smekens M, Hérin M, Coquette A (2004). A simple reconstructed human epidermis: preparation of the culture model and utilization in in vitro studies. Arch Dermatol Res.

[12] De Vuyst E, Charlier C, Giltaire S, De Glas V, de Rouvroit CL, Poumay Y (2014). Reconstruction of normal and pathological human epidermis on polycarbonate filter. Methods Mol Biol.

[13] Auxenfans C, Fradette J, Lequeux C, Germain L, Kinikoglu B, Bechetoille N (2009). Evolution of three dimensional skin equivalent models reconstructed in vitro by tissue engineering. Eur J Dermatol.

[14] Lancaster MA, Huch M. Disease modelling in human organoids. Dis Model Mech. 2019;12. 10.1242/dmm.039347. 10.1242/dmm.039347 PMC6679380 31383635

[15] Wang X, Wang S, Guo B, Su Y, Tan Z, Chang M (2021). Human primary epidermal organoids enable modeling of dermatophyte infections. Cell Death Dis.

[16] Ma J, Liu J, Gao D, Li X, Zhang Q, Lv L (2022). Establishment of human pluripotent stem cell-derived skin organoids enabled pathophysiological model of SARS-CoV-2 infection. Adv Sci (Weinheim, Baden-Wurttemberg, Ger.).

[17] Klevens RM, Morrison MA, Nadle J, Petit S, Gershman K, Ray S (2007). Invasive methicillin-resistant *Staphylococcus aureus* infections in the United States. JAMA.

[18] Talan DA, Krishnadasan A, Gorwitz RJ, Fosheim GE, Limbago B, Albrecht V (2011). Comparison of *Staphylococcus aureus* from skin and soft-tissue infections in US emergency department patients, 2004 and 2008. Clin Infect Dis Publ Infect Dis Soc Am.

[19] Shi C-S, Shenderov K, Huang N-N, Kabat J, Abu-Asab M, Fitzgerald KA (2012). Activation of autophagy by inflammatory signals limits IL-1β production by targeting ubiquitinated inflammasomes for destruction. Nat Immunol.

[20] Youn C, Archer NK, Miller LS (2020). Research techniques made simple: mouse bacterial skin infection models for immunity research. J Investig Dermatol.

[21] Kretzschmar K, Clevers H (2016). Organoids: modeling development and the stem cell niche in a dish. Dev Cell.

[22] Co JY, Margalef-Català M, Li X, Mah AT, Kuo CJ, Monack DM (2019). Controlling epithelial polarity: a human enteroid model for host-pathogen interactions. Cell Rep.

[23] Bartfeld S, Clevers H. Organoids as model for infectious diseases: culture of human and murine stomach organoids and microinjection of Helicobacter pylori. J Vis Exp. 2015. 10.3791/53359. 10.3791/53359 PMC4692704 26650279

[24] Bartfeld S, Bayram T, van de Wetering M, Huch M, Begthel H, Kujala P (2015). In vitro expansion of human gastric epithelial stem cells and their responses to bacterial infection. Gastroenterology.

[25] Co JY, Margalef-Català M, Monack DM, Amieva MR (2021). Controlling the polarity of human gastrointestinal organoids to investigate epithelial biology and infectious diseases. Nat Protoc.

[26] Bar-Ephraim YE, Kretzschmar K, Clevers H (2020). Organoids in immunological research. Nat Rev Immunol.

[27] Sachs N, Papaspyropoulos A, Zomer-van Ommen DD, Heo I, Böttinger L, Klay D, et al. Long-term expanding human airway organoids for disease modeling. EMBO J. 2019;38. 10.15252/embj.2018100300. 10.15252/embj.2018100300 PMC6376275 30643021

[28] Holokai L, Chakrabarti J, Broda T, Chang J, Hawkins JA, Sundaram N (2019). Increased programmed death-ligand 1 is an early epithelial cell response to Helicobacter pylori infection. PLoS Pathog.

[29] Neal JT, Li X, Zhu J, Giangarra V, Grzeskowiak CL, Ju J (2018). Organoid modeling of the tumor immune microenvironment. Cell.

[30] Li X, Ootani A, Kuo C (2016). An air-liquid interface culture system for 3D organoid culture of diverse primary gastrointestinal tissues. Methods Mol Biol.

[31] Driehuis E, Kretzschmar K, Clevers H (2020). Establishment of patient-derived cancer organoids for drug-screening applications. Nat Protoc.

[32] Nie X, Liang Z, Li K, Yu H, Huang Y, Ye L (2021). Novel organoid model in drug screening: past, present, and future. Liver Res.

[33] Zhou Z, Cong L, Cong X (2021). Patient-derived organoids in precision medicine: drug screening, organoid-on-a-chip and living organoid biobank. Front Oncol.

[34] Krüger J, Groß R, Conzelmann C, Müller JA, Koepke L, Sparrer KMJ (2021). Drug inhibition of SARS-CoV-2 replication in human pluripotent stem cell-derived intestinal organoids. Cell Mol Gastroenterol Hepatol.

[35] Ebisudani T, Sugimoto S, Haga K, Mitsuishi A, Takai-Todaka R, Fujii M (2021). Direct derivation of human alveolospheres for SARS-CoV-2 infection modeling and drug screening. Cell Rep.

[36] Groveman BR, Ferreira NC, Foliaki ST, Walters RO, Winkler CW, Race B (2021). Human cerebral organoids as a therapeutic drug screening model for Creutzfeldt-Jakob disease. Sci Rep.

[37] Han Y, Duan X, Yang L, Nilsson-Payant BE, Wang P, Duan F (2021). Identification of SARS-CoV-2 inhibitors using lung and colonic organoids. Nature.

[38] Dekkers JF, Alieva M, Wellens LM, Ariese HCR, Jamieson PR, Vonk AM (2019). High-resolution 3D imaging of fixed and cleared organoids. Nat Protoc.

[39] Wang M, Yu H, Zhang T, Cao L, Du Y, Xie Y (2022). In-depth comparison of matrigel dissolving methods on proteomic profiling of organoids. Mol Cell Proteom.

[40] Puschhof J, Pleguezuelos-Manzano C, Martinez-Silgado A, Akkerman N, Saftien A, Boot C (2021). Intestinal organoid cocultures with microbes. Nat Protoc.

[41] Bardou P, Mariette J, Escudié F, Djemiel C, Klopp C (2014). jvenn: an interactive Venn diagram viewer. BMC Bioinform.

