# Supplementary material for: Use of mouse primary epidermal organoids for USA300 infection modeling and drug screening
Source: Cell Death Dis. 2023 Jan 11;14(1):15. doi: 10.1038/s41419-022-05525-x (PMC9833019; doi:10.1038/s41419-022-05525-x)
Supplement: Supplementary file 2 — Supplementary Figure 1 [file 41419_2022_5525_MOESM2_ESM.docx]

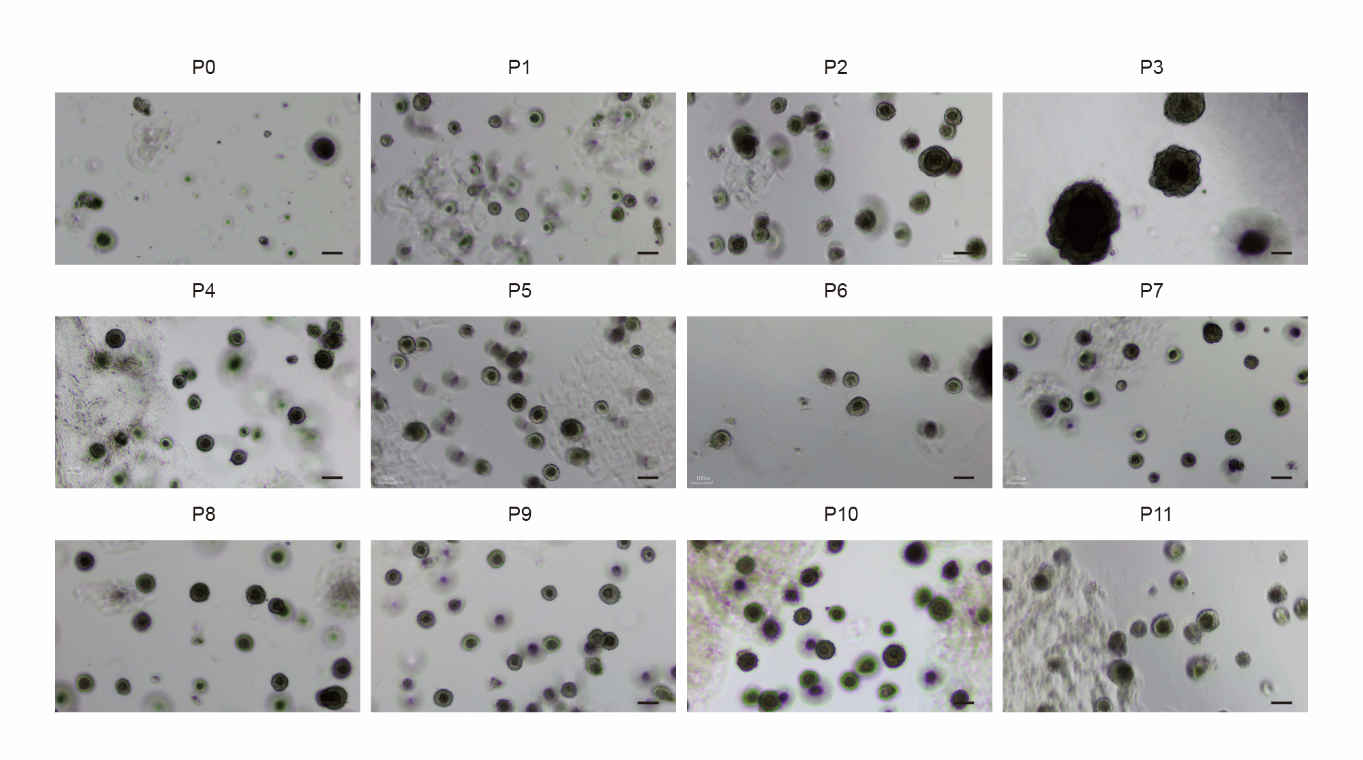
Supplementary Fig 1 Representative picture of mPEOs passing at a density of 1.25×10^5^/mL every 7 days. Scale bar=100μm.
